# Mitsugumin 53 regulates extracellular Ca^2+^ entry and intracellular Ca^2+^ release via Orai1 and RyR1 in skeletal muscle

**DOI:** 10.1038/srep36909

**Published:** 2016-11-14

**Authors:** Mi Kyoung Ahn, Keon Jin Lee, Chuanxi Cai, Mei Huang, Chung-Hyun Cho, Jianjie Ma, Eun Hui Lee

**Affiliations:** 1 Department of Physiology, College of Medicine, The Catholic University of Korea, 222 Banpo-daero, Seocho-gu, Seoul 06591, Republic of Korea; 2 Center for Cardiovascular Sciences, Department of Molecular and Cellular Physiology, Albany Medical College, 43 New Scotland Avenue, Albany, New York 12208, USA; 3 Department of Pharmacology, College of Medicine, Seoul National University, 103 Daehak-ro, Jongno-gu, Seoul 110-799, Republic of Korea; 4 Department of Surgery, Davis Heart and Lung Research Institute, The Ohio State University, 360 W. 12th Ave, Columbus, Ohio 43210, USA

## Abstract

Mitsugumin 53 (MG53) participates in the membrane repair of various cells, and skeletal muscle is the major tissue that expresses MG53. Except for the regulatory effects of MG53 on SERCA1a, the role(s) of MG53 in the unique functions of skeletal muscle such as muscle contraction have not been well examined. Here, a new MG53-interacting protein, Orai1, is identified in skeletal muscle. To examine the functional relevance of the MG53-Orai1 interaction, MG53 was over-expressed in mouse primary or C2C12 skeletal myotubes and the functional properties of the myotubes were examined using cell physiological and biochemical approaches. The PRY-SPRY region of MG53 binds to Orai1, and MG53 and Orai1 are co-localized in the plasma membrane of skeletal myotubes. MG53-Orai1 interaction enhances extracellular Ca^2+^ entry via a store-operated Ca^2+^ entry (SOCE) mechanism in skeletal myotubes. Interestingly, skeletal myotubes over-expressing MG53 or PRY-SPRY display a reduced intracellular Ca^2+^ release in response to K^+^-membrane depolarization or caffeine stimulation, suggesting a reduction in RyR1 channel activity. Expressions of TRPC3, TRPC4, and calmodulin 1 are increased in the myotubes, and MG53 directly binds to TRPC3, which suggests a possibility that TRPC3 also participates in the enhanced extracellular Ca^2+^ entry. Thus, MG53 could participate in regulating extracellular Ca^2+^ entry via Orai1 during SOCE and also intracellular Ca^2+^ release via RyR1 during skeletal muscle contraction.

Skeletal muscle contraction is accomplished by the operation of excitation-contraction (EC) coupling^1^,^2^,^3^,^4^,^5^. During skeletal EC coupling, acetylcholine receptors in the plasma membrane of skeletal muscle cells are activated by acetylcholine released from a motor neuron, and Na^+^ influx through the activated acetylcholine receptors induces membrane depolarization. Membrane depolarization induces muscle action potential in skeletal muscle cells, and the action potential spreads along the surface of the plasma membrane and to the interior of skeletal muscle cells via transverse (t)-tubule invaginations. The spreading of the action potential activates dihydropyridine receptors (DHPR, a Ca^2+^ channel on the t-tubule membrane), which, in turn, activates ryanodine receptor 1 (RyR1, a Ca^2+^ channel on sarcoplasmic reticulum (SR) membrane) via physical interactions between DHPR and RyR1. This results in the release of Ca^2+^ ions from the SR to the cytosol via RyR1 and skeletal muscle contraction by the binding of the released Ca^2+^ ions to contractile proteins. Ca^2+^ ions also activate RyR1 by binding to RyR1, which is called Ca^2+^-induced Ca^2+^ release (CICR), and CICR in skeletal myotubes plays a role in maximizing and maintaining the Ca^2+^ supply for skeletal muscle contraction^1^,^5^. Extracellular Ca^2+^ entry, such as store-operated Ca^2+^ entry (SOCE) via Orai1 or canonical-type transient receptor potential cation channels (TRPC), partially contributes to the Ca^2+^ supply for skeletal muscle contraction^6^,^7^,^8^,^9^. Orai1 is a major Ca^2+^ channel responsible for SOCE in skeletal muscle^3^,^10^. During skeletal muscle relaxation, sarcoplasmic/endoplasmic reticulum Ca^2+^-ATPase 1a (SERCA1a) uptakes Ca^2+^ from the cytosol to the SR in order to reduce cytosolic Ca^2+^ to its resting level and to replenish the SR with Ca^2+^ ions^3^,^11^. An efficient arrangement of the proteins mentioned above is maintained by the junctional membrane complexes where t-tubule and the SR membranes are closely juxtaposed^4^,^12^,^13^.

Mitsugumin 53 (MG53, also called TRIM72) is expressed in skeletal and cardiac muscle, in lungs, and in kidneys, but skeletal muscle is the major site for MG53 expression^14^,^15^,^16^. MG53 is a tripartite motif (TRIM) family protein and is composed of a TRIM domain at the N-terminus and a PRY domain (a domain associated with SPRY) followed by a SPRY domain (a sequence repeat in the dula-specificity kinase splA and ryanodine receptor) at the C-terminus^16^,^17^,^18^,^19^,^20^. The TRIM domain is sub-divided into a ring domain harboring ubiquitin E3 ligase activity, a b-box harboring a zinc-binding moiety, and two coiled-coil domains^17^,^18^. Together with dysferlin, polymerase I and transcript release factor (PTRF), and non-muscle myosin type IIA, MG53 constitutes the membrane repair system^16^,^19^,^21^,^22^,^23^. MG53 protein coats intracellular vesicles via binding to phosphatidylserine on the membranes of intracellular vesicles^16^. During injury, oligomerization of MG53 through oxidation of the thiol group of cysteine at 242 and a leucine zipper motif between the two coiled-coil domains induces nucleation of the intracellular vesicles coated with MG53, the trafficking of the vesicles to the injury sites, and the resealing of injured membranes^16^,^24^. The binding of caveolin 3 to MG53 moderates the robust vesicle trafficking to the injury sites^19^,^23^. MG53 knockout mice show the progressive skeletal myopathy that is associated with defective membrane repair and the increased vulnerability of cardiomyocytes to ischemia-reperfusion-induced injury^16^,^25^,^26^. By enhancing membrane repair, MG53 ameliorates the pathology of skeletal muscular dystrophy in a hamster model^27^ as well as that of Duchenne Muscular Dystrophy in a mdx mouse model^28^. Purified MG53 has cardio-protective effects against myocardial infarction^29^. In addition to skeletal and cardiac muscle cells, MG53 exists in the bloodstream and plays a protective role against tissue injuries such as acute lung or kidney injury^14^,^15^,^28^.

Apart from the roles of MG53 in the membrane repair system, there have been a few reports on the roles of MG53 in the unique functions of skeletal muscle, such as contraction and relaxation. MG53 facilitates the differentiation of C2C12 skeletal myoblasts to myotubes by enhancing vesicle trafficking and membrane fusion^19^. MG53 attenuates SERCA1a activity during skeletal muscle contraction by the binding of its TRIM and PRY domains to SERCA1a, and contributes to more efficient skeletal muscle contraction^30^. Considering that MG53 ameliorates skeletal muscle diseases such as skeletal muscle dystrophy/myopathy^16^,^23^,^27^,^28^, it is possible that MG53 plays other important roles in skeletal muscle contraction and/or relaxation. Therefore, in the present study, we attempted to find MG53-interacting proteins with their binding sites on MG53, and to examine the roles of MG53 along with the MG53-interacting proteins in Ca^2+^ movements in skeletal muscle using mouse primary skeletal myotubes, rabbit skeletal muscle tissue, a C2C12 mouse skeletal muscle cell line, and biochemical and cell physiological approaches.

## Results

### MG53 binds to Orai1 via its PRY-SPRY region and co-localizes with Orai1 in the plasma membrane in skeletal muscle

**Figure 1:**
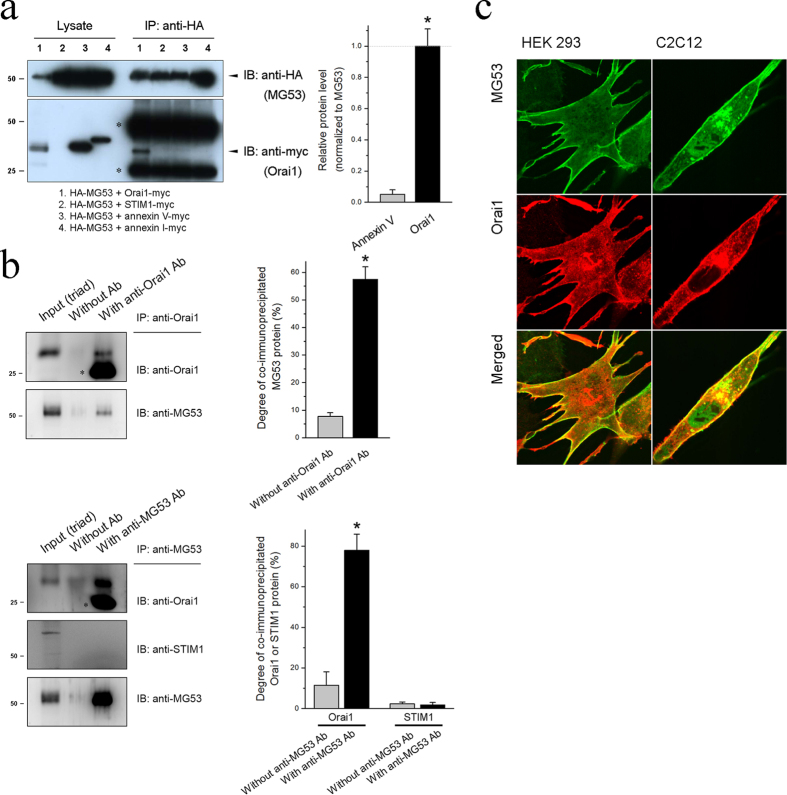
Interaction between MG53 and Orai1. (**a**) HEK293 cells were co-transfected with constructs of HA-MG53 along with Orai1-myc, STIM1-myc, annexin V-myc, or annexin I-myc. 24 h after the transfection, cell lysates were immunoprecipitated with anti-HA antibody, and were immunobloted with anti-HA or anti-myc antibodies. Three independent experiments were conducted. Lysate refers to a simple immunoblot, and IP to immunoprecipitation. The asterisks indicate the artifact band for the heavy or light chains of anti-HA antibody (about 50 or 25 kDa) due to the cross-reactivity of the anti-myc antibody to the chains. Quantitative analysis for the band intensity of co-immunoprecipitated Orai1 with MG53 is presented in the bar graphs in the right-hand panel. Annexin V was used as a negative control, and the normalized value to MG53 was normalized to those of Orai1. *Significant difference compared with Annexin V (*p* < 0.05). (**b**) The triad vesicle sample obtained from rabbit skeletal muscle (30 μg of total proteins) was subjected to co-immunoprecipitation assay with anti-Orai1 (upper panel) or anti-MG53 antibody (lower panel), and was immunobloted with anti-Orai1, anti STIM1, or anti-MG53 antibodies. Input (triad) indicates the simple immunoblot of the triad vesicle sample (5 μg of total proteins). Without Ab indicates a reaction without anti-Orai1 or anti-MG53 antibody. Three independent experiments per each were conducted. The reaction without anti-Orai1 or anti-MG53 antibody was used as a negative control. The asterisks in blots indicate the artifact band for the light chains of anti-Orai1 or anti-MG53 antibody (about 25 kDa) due to the cross-reactivity of the anti-Orai1 antibody to the chains. Full-length blots are presented in Supplementary Figs 1 and 2. Degree of co-immunoprecipitated MG53 to total MG53 (upper panel) or degree of co-immunoprecipitated Orai1 or STIM1 to the corresponding total protein (lower panel) is presented in the bar graphs in the right-hand panel. *Significant difference compared with Without anti-Orai1 Ab or Without anti-MG53 Ab (*p* < 0.05). (**c**) HEK293 cells (left-hand panel) or C2C12 mouse skeletal myotubes (right-hand panel) co-transfected with GFP-MG53 and Orai1-myc constructs were subjected to immunocytochemistry with anti-GFP and anti-myc antibodies. The data are representative images from three independent experiments (ten images for each group).

To examine the interaction of MG53 with SOCE-mediating proteins such as Orai1, HEK293 cells were co-transfected with HA-MG53 and Orai1-myc constructs, and the cell lysate was subjected to a co-immunoprecipitation assay using anti-HA antibodies (Fig. 1a). Annexin proteins bind to the negatively charged phospholipids of membranes^31^, and the cell lysate obtained from HEK293 cells transfected with annexin V-myc or annexin I-myc construct was used as a negative control for the assay (lanes labeled 3 or 4 in Fig. 1a). Orai1 was successfully co-immunoprecipitated with MG53 (lane 1). However, STIM1, a major Orai1-activating/regulating protein, did not bind to MG53 (lane 2). To have confidence in the data, we conducted co-immunoprecipitation assays using the triad vesicle sample from the rabbit skeletal muscle which is a *‘bona fide*’ tissue to express MG53, Orai1, and STIM1, and with anti-Orai1 (Fig. 1b, upper panel) or anti-MG53 antibody (Fig. 1b, lower panel) (Supplementary Figs 1 and 2). MG53 was also co-immunoprecipitated with Orai1 (and vice versa) but not with STIM1. Therefore, based on the two different approaches (heterologous expression in HEK293 cells and rabbit skeletal muscle tissue), we suggest that MG53 binds to Orai1 in skeletal muscle.

Immunocytochemistry of HEK293 cells expressing HA-MG53 and Orai1-myc using anti-HA and anti-myc antibodies showed the co-localization of MG53 and Orai1 in the plasma membrane (Fig. 1c, lower panel). In addition, the co-localization of MG53 and Orai1 in the plasma membrane was also found in C2C12 myotubes that were differentiated forms of a mouse skeletal myoblast cell line (Fig. 1c, bottom panel). These results reveal that MG53 displays a plasma membrane co-localization pattern with Orai1 in skeletal muscle.

**Figure 2:**
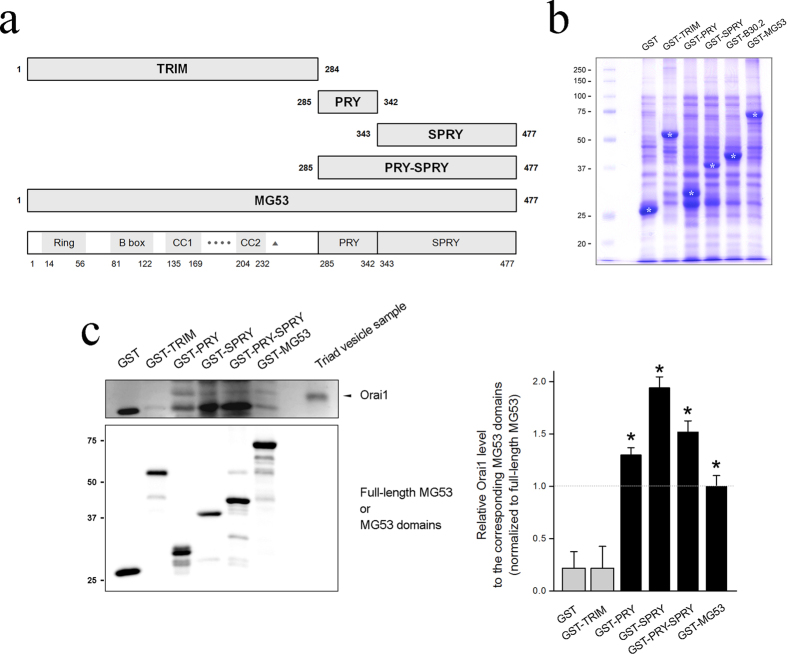
Binding of the PRY-SPRY region of MG53 to Orai1. (**a**) Schematic diagrams of full-length MG53 and MG53 domains. Numbers indicate the sequence of amino acids. TRIM, tripartite motif; CC, coiled-coil; four black dots, a zinc-binding leucine zipper motif; a triangle, a cysteine between the two CC domains; PRY, a domain associated with SPRY domains^50^; SPRY, a sequence repeat in the dula-specificity kinase splA and ryanodine receptor^51^. (**b**) Various GST-MG53 domains expressed in *E. coli* (indicated by white asterisks) were separated on a SDS–PAGE gel (10%) and were stained with Coomassie Brilliant Blue staining. (**c**) The bound proteins obtained from the binding assays of GST-MG53 domains with the triad vesicle sample from rabbit skeletal muscle were separated on SDS–PAGE gels (12%) and were subjected to immunoblot assays with anti-Orai1 or anti-GST-antibodies. GST was used as a negative control. Three independent experiments were conducted. Full-length blots are presented in Supplementary Fig. 3. The relative amount of Orai1 to the corresponding amount of MG53 domain is presented in bar graphs in the right-hand panel. The value for the relative amount of Orai1 to full-length MG53 (GST-MG53) was regarded as 1, and others were normalized by this value. *Significant difference compared with GST control (*p* < 0.05).

To find Orai1-binding region(s) in MG53, cDNAs for four GST-fused MG53 domains were constructed (Fig. 2a): GST-TRIM, GST-PRY, GST-SPRY, and GST-PRY-SPRY, along with GST-full-length MG53. Each GST-fused MG53 domain was expressed in *E. coli*, and the bacterial cell lysates were separated on a SDS-PAGE gel and were stained with Coomassie Brilliant Blue (Fig. 2b). All GST-fused MG53 proteins were successfully expressed. For binding assays of MG53 domains with Orai1, affinity beads were prepared by immobilizing each GST-fused MG53 protein on GST beads, and the affinity beads were incubated with the triad vesicle sample, an enriched portion with triad proteins that mediate intra and extracellular Ca^2+^ movements including Orai1, from rabbit skeletal muscle^3^,^4^,^32^. The GST-pull down samples were immunobloted with anti-Orai1 or anti-GST antibodies (Fig. 2c, left-hand panel, Supplementary Fig. 3). The relative amounts of Orai1 and MG53 in the binding assays are presented as bar graphs in the right-hand panel. As expected based on Fig. 1a,b, full-length MG53 was bound to Orai1 (Fig. 2c). PRY, SPRY, and PRY-SPRY (all domains except for TRIM domain) were also bound to Orai1, suggesting that there are binding sites for Orai1 in both the PRY and SPRY domains on MG53 and that binding of Orai1 to either domain is as effective as binding to both. Therefore, the PRY-SPRY region of MG53 was responsible for the binding to Orai1.

### MG53-Orai1 interaction in skeletal myotubes enhances extracellular Ca^2+^ entry via Orai1 by SOCE mechanism

**Figure 3:**
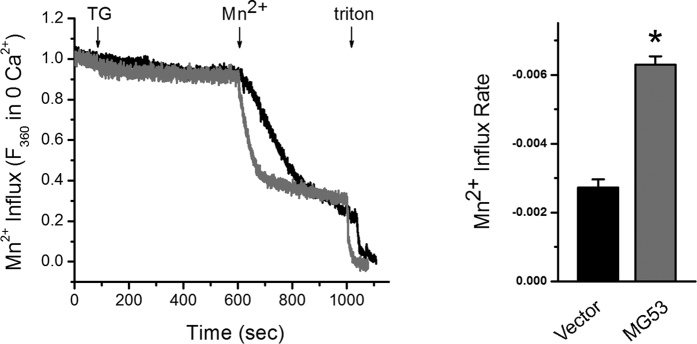
Enhanced SOCE by MG53. C2C12 skeletal myotubes over-expressing MG53 were loaded with 5 μM fura-2-AM, and the SR Ca^2+^ storage was depleted with the addition of TG (2.5 μM) in the absence of extracellular Ca^2+^. The addition of extracellular Mn^2+^ (0.5 mM) led to the quenching of intracellular fura-2 fluorescence. A representative trace for each group is shown. The rate of Mn^2+^ influx was presented as bar graphs in the right-hand panel. The rate of Mn^2+^ influx was determined from the variable (i.e., slope) of a linear equation obtained from a linear fitting of the traces from the initial 10 seconds. A steeper slope indicates a more active SOCE. The results are presented as the mean ± S.E. of six (for Vector) or eight independent experiments (for MG53). Triton was used as an indicator that cell membranes are intact. *Significant difference compared with Vector controls (*p* < 0.05).

**Figure 4:**
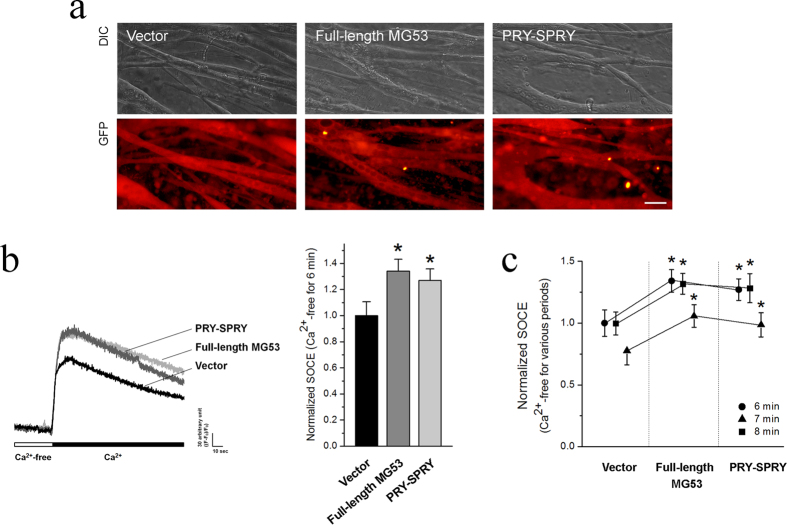
Enhanced SOCE by full-length MG53 or PRY-SPRY. (**a**) Expression of full-length MG53 or PRY-SPRY in mouse primary skeletal myotubes was visualized by immunocytochemistry. Vector indicates GFP alone. Bar represents 50 μm. (**b**) The SR Ca^2+^ storage of myotubes over-expressing full-length MG53 or PRY-SPRY was depleted by the treatment of TG (2.5 μM) in the absence of extracellular Ca^2+^. Extracellular Ca^2+^ (2 mM) was applied to the myotubes to induce SOCE. A representative trace for each group is shown, and the results are summarized as bar graphs in the right-hand panel. *Significant difference compared with Vector controls (P < 0.05). The values are presented as the mean ± S.E. for the number of myotubes shown in the parentheses of Table 1. (**c**) Three different time intervals between the TG treatment (Ca^2+^ depletion from SR) and extracellular Ca^2+^ application (SOCE) were applied to the myotubes. The values were normalized to the mean value of those from vector controls at Ca^2+^-free for 6 min. The results are presented as the mean ± S.E. for the number of myotubes shown in the parenthesis in Table 1. *Significant difference versus corresponding Vector control (*p* < 0.05).

Our lab previously established a method for the quantification of SOCE in skeletal muscle cells, using Mn^2+^-quenching of intracellular fura-2 fluorescence^33^,^34^,^35^. Mn^**2+**^ is known to permeate cells via a SOCE mechanism but is insensitive to both the surface membrane extrusion processes and SR uptake by Ca^2+^ pumps. When Mn^2+^ binds to intracellular fura-2, it quenches the fura-2 fluorescence measured at excitation wavelength of 360 nm. Thus, the rate of Mn^2+^ fluorescence quenching (i.e., the rate of Mn^2+^ influx) represents a measurement of the unidirectional Ca^2+^ entry. To examine the functional relevance of MG53 in skeletal SOCE, MG53 was over-expressed in C2C12 skeletal myotubes. The SR Ca^2+^ storage of the myotubes loaded with fura-2 was depleted with the addition of thapsigargin (TG) in the absence of extracellular Ca^2+^, and the addition of extracellular Mn^2+^ led to a quenching of intracellular fura-2 fluorescence (i.e., Mn^2+^-quenching experiment reflecting SOCE) (Fig. 3). C2C12 skeletal myotubes over-expressing MG53 showed a significantly faster rate of Mn^2+^ influx compared with vector controls, suggesting that MG53 could enhance SOCE in skeletal muscle. In a separate manner, full-length MG53 was over-expressed in mouse primary skeletal myotubes, and the successful expression of the full-length MG53 was confirmed by immunocytochemistry (Fig. 4a). To induce SOCE, the SR Ca^2+^ storage of mouse primary skeletal myotubes over-expressing full-length MG53 was depleted with TG in the absence of extracellular Ca^2+^, and extracellular Ca^2+^ was applied to the myotubes (Fig. 4b). MG53 in mouse primary skeletal myotubes also significantly enhanced SOCE compared with the vector control (Table 1). To examine the role of MG53-Orai1 interaction in the enhanced SOCE by full-length MG53, PRY-SPRY (i.e., Orai1-binding region of MG53) was over-expressed in mouse primary skeletal myotubes (Fig. 4a), and the same experiments were conducted with the myotubes (Fig. 4b). Enhancements in SOCE were also observed in the myotubes over-expressing PRY-SPRY, as shown by full-length MG53 (Fig. 4b and Table 1). As Orai1 is the major Ca^2+^ channel responsible for SOCE in skeletal muscle^3^,^10^, these results provide further evidence that binding of the PRY-SPRY domain of MG53 to Orai1 modulates the activity of SOCE. Three different time intervals between the TG treatment (Ca^2+^ depletion from SR) and extracellular Ca^2+^ application (SOCE) were applied to the myotubes (Fig. 4c and Table 1). Enhancements in SOCE by full-length MG53 or PRY-SPRY were maintained during all durations.

**Figure 5:**
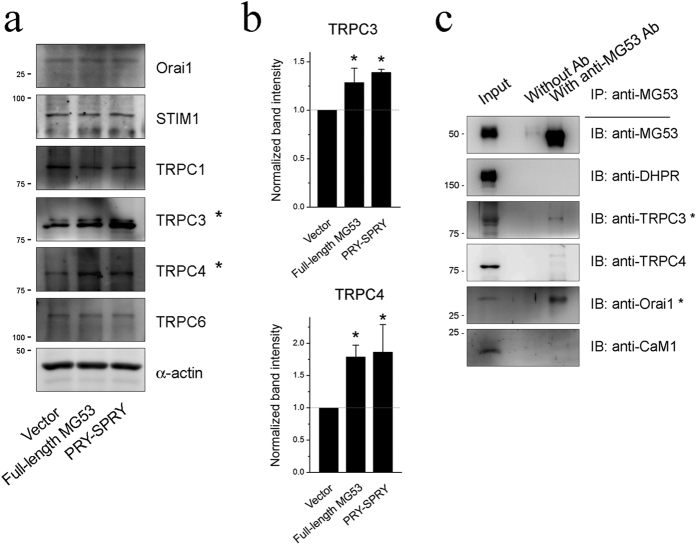
Increased expression levels of TRPC3 and TRPC4 by full-length MG53 or PRY-SPRY, and the binding of TRPC3 to MG53. (**a**) Lysate from myotubes over-expressing full-length MG53 or PRY-SPRY was subjected to an immunoblot assay with one of the antibodies against six proteins that are known to be expressed and/or to mediate extracellular Ca^2+^ entry into skeletal muscle. *α*-Actin was used as a loading control. Three independent experiments per each protein were conducted. (**b**) The expression levels of TRPC3 and TRPC4 (indicated by asterisks in a) were presented as bar graphs. Bar graphs were presented as the mean ± S.E. for three independent experiments. *Significant difference versus corresponding Vector control (*p* < 0.05). Full-length blots are presented in Supplementary Figs 4 to 10. (**c**) The triad vesicle sample obtained from rabbit skeletal muscle (30 μg of total proteins) was subjected to a co-immunoprecipitation assay with anti-MG53 antibody, and the immunoprecipitant was subjected to immunoblot analysis with various antibodies. Input indicates the simple immunoblot of the triad vesicle sample (5 μg of total proteins). “Without Ab” indicates a reaction without anti-MG53 antibody. TRPC3 was co-immunoprecipitated with MG53 (indicated by an asterisk). Three independent experiments were conducted. IB or IP means immunoblot or immunoprecipitation. CaM1 refers to calmodulin1. Full-length blots are presented in Supplementary Fig. 11.

To examine other proteins related to the enhanced SOCE by the MG53-Orai1 interaction, expression levels of proteins known to express and/or mediate extracellular Ca^2+^ entry (TRPC1, TRPC3, TRPC4, and TRPC6 along with Orai1 and STIM1) were evaluated via immunoblot assays using the lysate of myotubes over-expressing MG53 or PRY-SPRY (Fig. 5a, Supplementary Figs 4 to 10). The major SOCE-mediating proteins, Orai1 and STIM1 were not changed in their expression levels, suggesting that the enhanced SOCE by the MG53-Orai1 interaction is due to the increase in the channel activity of Orai1 rather than to the increase in the expression of Orai1 or STIM1. Interestingly, TRPC3 and TRPC4 were up-regulated in their expressions (Fig. 5a,b). These results suggest the possibility that, in addition to Orai1, TRPC3 and TRPC4 also contribute to the enhanced SOCE by the MG53-Orai1 interaction. To examine this possibility, co-immunoprecipitation assays of MG53 were conducted with both TRPC3 and TRPC4 (Fig. 5c, Supplementary Fig. 11), and the interaction of MG53 with DHPR was also examined. Among them, TRPC3 was co-immunoprecipitated with MG53. Therefore, it is possible that, at least, TRPC3 is directly related to the enhancement of SOCE by the MG53-Orai1 interaction, but DHPR, and TRPC4 are not.

### Skeletal myotubes over-expressing MG53 show reduced intracellular Ca^2+^ release via RyR1

**Figure 6:**
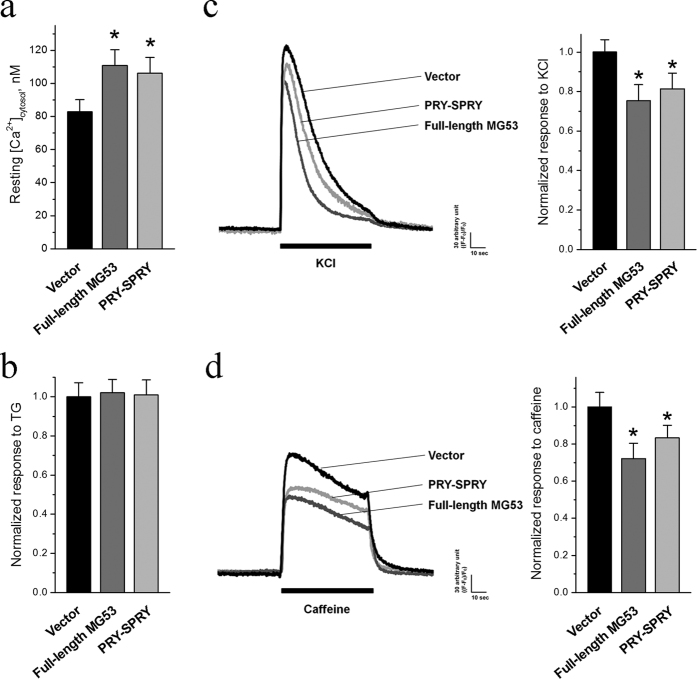
The increased resting cytosolic Ca^2+^ levels and the reduced response to KCl or caffeine by full-length MG53 or PRY-SPRY. (**a**) The resting cytosolic Ca^2+^ levels in the myotubes over-expressing full-length MG53 or PRY-SPRY were measured. (**b**) To measure the releasable Ca^2+^ from the SR to the cytosol, TG (2.5 μM) was applied to the myotubes in the absence of extracellular Ca^2+^. The results are presented as the mean ± S.E. for the number of myotubes shown in the parenthesis in Table 1. KCl that is a membrane depolarizer and induces skeletal muscle contraction (**c**), or caffeine that is a specific and direct RyR1agonist (**d**), was applied to the myotubes over-expressing full-length MG53 or PRY-SPRY. A representative trace for each group is shown, and bar graphs of the peak amplitude normalized to the mean value of those from the vector controls are shown in the right-hand panels. The results are presented as the mean ± S.E. for the number of myotubes shown in the parenthesis in Table 1. *Significant difference versus Vector control (*p* < 0.05).

The enhancement of SOCE by the MG53-Orai1 interaction may impact the intracellular Ca^2+^ homeostasis and signaling in skeletal muscle. The resting cytosolic Ca^2+^ levels in skeletal myotubes over-expressing MG53 or PRY-SPRY were quantified using fura-2 ratiometric measurement. As shown in Fig. 6a, the resting cytosolic Ca^2+^ level was significantly increased by the over-expression of full-length MG53 or RPY-SPRY compared with that in myotubes transfected with vector control, reflecting the enhanced SOCE activity. Interestingly, when the TG-releasable Ca^2+^ content from the SR in the absence of extracellular Ca^2+^ was assayed, there was no significant difference in the total SR Ca^2+^ content in skeletal myotubes transfected with the full-length MG53, PRY-SPRY, or vector control (Fig. 6b). Our previous study identified the functional interaction between MG53 and SERCA1a that contributes to the modulation of Ca^2+^ recycling into the SR^30^. Thus, MG53-mediated suppression of SERCA1a activity could account for the balanced SR Ca^2+^-handling, even in the presence of elevated cytosolic Ca^2+^ levels.

The functional properties of myotubes over-expressing full-length MG53 or PRY-SPRY were also examined by measuring intracellular Ca^2+^ release in the presence of a membrane depolarizer, KCl (that induces membrane depolarization and induces the coupling between DHPR and RyR1 and Ca^2+^ release from the SR to cytosol via RyR1 for skeletal muscle contraction^1^,^3^,^4^,^5^, Fig. 6c and Table 1). Surprisingly, responses to KCl were decreased in the myotubes over-expressing full-length MG53, and similar responses were found in the myotubes over-expressing PRY-SPRY. In a separate assay, caffeine, a specific and direct agonist of RyR1, was applied to the myotubes (Fig. 6d and Table 1). Responses to caffeine were also decreased approximately equal to the responses to KCl in the myotubes over-expressing full-length MG53 or PRY-SPRY. Therefore, these results suggest a reduction in Ca^2+^ release from the SR via RyR1 in myotubes with the over-expression of full-length MG53 or PRY-SPRY. The reduced Ca^2+^ release from the SR via RyR1 was not due to a simple decrease in the amount of Ca^2+^ stored in the SR, because there was no significant change in the total releasable Ca^2+^ from the SR (Fig. 6b).

**Figure 7:**
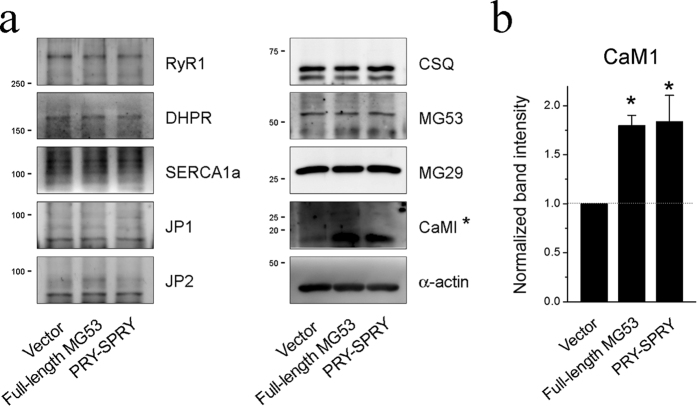
Increased expression level of CaM1 by full-length MG53 or PRY-SPRY. (**a**) Lysate from the myotubes over-expressing full-length MG53 or PRY-SPRY was subjected to an immunoblot assay with one of the antibodies against nine proteins that are known to mediate skeletal EC coupling and/or the handling of Ca^2+^. *α*-Actin was used as a loading control. Three independent experiments per each protein were conducted. JP, junctophilin; CSQ, calsequestin. (**b**) Among them, the expression level of CaM1 (indicated by asterisks in a) is presented as bar graphs. Bar graphs are presented as the mean ± S.E. for three-independent experiments. *Significant difference versus Vector control (*p* < 0.05). Full-length blots are presented in Supplementary Figs 12 to 21.

We conducted additional biochemical assays to determine whether the over-expression of full-length MG53 or PRY-SPRY can alter the protein components of skeletal EC coupling machinery. Figure 7a (Supplementary Figs 12 to 21) shows immunoblot assays using the lysate of myotubes over-expressing full-length MG53 or PRY-SPRY. There was no significant change in the expression levels of three main proteins that mediate Ca^2+^ movements between the SR and cytosol during skeletal muscle contraction or relaxation: DHPR, RyR1, and SERCA1a. There was also no change in the expression levels of proteins that mediate the formation of the junctional membrane complex and the handling of Ca^2+^: junctophilin 1 (JP1), JP2, calsequestrin, and mitsugumin 29. However, the expression level of calmodulin 1 (CaM1), a Ca^2+^-dependent protein, was significantly up-regulated by the over-expression of full-length MG53 or PRY-SPRY (Fig. 7b). Previous studies by Seiler *et al*. and Tripathy *et al*. showed that Ca^2+^-charged CaM1 (Ca^2+^-CaM1) exerts an inhibitory effect on RyR1 channel activity^36^,^37^. Thus, compensatory changes in Ca^2+^-CaM1 interaction with RyR1 may contribute to the reduced Ca^2+^ release via RyR1 in myotubes with the over-expressions of either MG53 or PRY-SPRY. In addition, it is not likely that CaM1 itself directly affects either MG53 or the enhanced SOCE via the MG53-Orai1 interaction, because there was no direct interaction between MG53 and CaM1 (Fig. 5c).

## Discussion

In the present study, we found that MG53 in skeletal muscle binds to Orai1 via its PRY-SPRY region. Apart from the binding of MG53 to phosphatidylserine on the plasma membranes or intracellular vesicles for the repair of membrane injuries, MG53 shows plasma membrane co-localization with Orai1. The MG53-Orai1 interaction enhances extracellular Ca^2+^ entry via Orai1, and decreases intracellular Ca^2+^ release via RyR1 for skeletal muscle contraction. In addition, increases in the expressions of TRPC3, TRPC4, and CaM1 are also found, and TRPC3 possibly participates in the enhancement of extracellular Ca^2+^ entry by binding directly to MG53.

Previously, we reported that the TRIM and PRY domains of MG53 constitute the binding region to SERCA1a, and MG53-SERCA1a interaction attenuates the activity of SERCA1a^30^ that uptakes Ca^2+^ from the cytosol to the SR in order to reduce cytosolic Ca^2+^ levels to the resting level during skeletal muscle relaxation^1^,^4^,^38^. In the present study, PRY and SPRY domains of MG53 constitute the binding region to Orai1, and MG53-Orai1 interaction in skeletal muscle enhances SOCE and cytosolic Ca^2+^ levels. Interestingly, these two different processes in skeletal muscle share a common PRY domain, and the PRY domain of MG53 could be a protein interface for the protein-protein interactions of MG53 with other proteins, as well as a protein module to regulate Ca^2+^ movements in skeletal muscle. This suggests that MG53 could play significant and various roles in the Ca^2+^ movements of skeletal muscle. We showed that the MG53-Orai1 interaction-mediated enhancement of SOCE activity leads to an elevation in resting cytosolic Ca^2+^ levels. Even with an elevated cytosolic Ca^2+^ level, there was no detectable overload of SR Ca^2+^ storage, which likely reflects the suppression of SERCA1a activity by MG53.

The three-dimensional structures of the PRY-SPRY domain of MG53 (i.e., the Orai1-binding region on MG53) have been revealed (GenBank accession number: NM_001008274.3): PRY-SPRY domain forms a binding pocket that is significantly broader and deeper^20^ and is sufficient to serve as the binding region to Orai1.

The knock-down of MG53 in skeletal muscle increases SERCA1a activity^30^. STIM1 has the opposite effect — its knock-down reduces SERCA1a activity^39^. However, both MG53 and STIM1 increases SOCE in skeletal muscle, and it seems that the regulation of SOCE by MG53 is different from that by STIM1, because MG53 over-expression involves an increase in resting cytosolic Ca^2+^ levels (in the present study), while STIM1 over-expression does not^39^. Therefore, it is possible that, in addition to MG53-Orai1 interaction (i.e., the increase of Orai1 activity by MG53), the enhanced SOCE involves other assistant proteins such as TRPC3, as shown in Fig. 5c. There are several reports on the participation of TRPC3 in SOCE; heteromerized TRPC3 with Orai1 mediates SOCE in HEK293 cells^40^, and adult myocytes isolated from TRPC3 transgenic mice show an increased SOCE^41^. TRPC3 contributes to the maintenance of the resting cytosolic Ca^2+^ levels of skeletal muscle^42^. Therefore, the increased expression of TRPC3 and the binding of TRPC3 to MG53 could be related to the enhanced SOCE and resting cytosolic Ca^2+^ by the MG53-Orai1 interaction, suggesting that TRPC3 could serve not only as proteins mediating SOCE^3^,^4^,^8^,^43^, but also could take part in regulating Ca^2+^ movements for skeletal EC coupling.

Ca^2+^-CaM1 inhibits Ca^2+^ release via RyR1 at micromolar cytosolic Ca^2+^ concentrations in skeletal muscle, and the inhibition is independent of the kinase activity of CaM1^36^,^37^. In the present study, the enhanced SOCE by the MG53-Orai1 interaction could induce a switch of aop-CaM1 to Ca^2+^-CaM1 that can inhibit RyR1 channel activity. Therefore, it is possible that, as a compensatory mechanism against extreme SOCE by the MG53-Orai1 interaction, Ca^2+^-CaM1 down-regulates RyR1 activity in order to regulate cytosolic Ca^2+^ levels. This is well supported by a previous report that CaM1 participates in regulating cytosolic Ca^2+^ levels by regulating Ca^2+^ release from the SR in skeletal muscle at elevated cytosolic Ca^2+^ levels^37^. According to the hypothesis, CaM1 and MG53 have a yin-and-yang type relationship (i.e., negative and positive) in regulating resting cytosolic Ca^2+^ levels in skeletal muscle, and CaM1 could be a functional competitor of MG53 in regulating Ca^2+^ homeostasis in skeletal muscle.

## Methods

### Ethics statement

All surgical interventions, including pre- and post-surgical animal care, were carried out in accordance with the Laboratory Animals Welfare Act, the Guide for the Care and Use of Laboratory Animals, and the Guidelines and Policies for Rodent Survival Surgery approved by the Institutional Animal Care and Use Committee of the College of Medicine at The Catholic University of Korea.

### DNA construction and the protein expression of GST-fused MG53 domains

Using full-length mouse MG53 DNA (GenBank accession number: NM_001079932) as a template, GST-fused full-length MG53 or MG53 domains were constructed and were expressed in *E. coli* (DH5α), as previously described^30^,^44^. GFP-tagged full-length MG53 or PRY-SPRY was constructed using an eGFP vector, as previously described^16^. HA-MG53 was constructed, as previously described^23^. Orai1-myc was constructed by inserting Orai1 DNA (GenBank accession number: NM_175423.3) into the 5′ end of pcDNA3.1 A containing a myc-tag (Invitrogen, Waltham, MA, USA)^23^.

### Preparation of triad vesicles and the binding assay of GST-fused MG53 domains with triad proteins

The triad vesicles enriched with triad proteins that mediate intra and extracellular Ca^2+^ movement in skeletal muscle, which included Orai1^3^,^4^,^32^ were prepared and solubilized to create a triad vesicle sample, as previously described^32^,^39^,^45^. Binding assays were performed, as previously described^30^,^44^.

### Cell culture and DNA transfection

Mouse primary skeletal myoblasts were derived from mouse skeletal muscle using a single-cell cloning method, and then were proliferated and differentiated to myotubes, as previously described^12^,^32^,^39^,^46^,^47^,^48^. For the differentiation of primary skeletal myoblasts into myotubes, the myoblasts were re-plated either on 10-cm plates (for the preparation of myotube lysates) or on 96-well plates (for the single-myotube Ca^2+^ imaging experiment) coated with Matrigel (BD Biosciences, San Jose, CA, USA). For the expression of GFP-tagged full-length MG53 or PRY-SPRY, immature primary skeletal myotubes on differentiation day 3 were transfected with each DNA construct using FuGENE6 transfection reagent (Promega, Madison, WI, USA). The C2C12 mouse skeletal myoblast cell line (American Type Culture Collection, Manassas, VA) was grown and differentiated to myotubes, as previously described^19^,^23^. HEK293 cells were grown at 37 °C in a 5% CO_2_ incubator in high DMEM with 10% FEB, 100 units/ml of penicillin, and 100 μg/ml of streptomycin^8^. For the expressions of GFP-, HA-, or myc-tagged proteins, C2C12 mouse skeletal myotubes or HEK293 cells on glass-bottomed dishes were transfected with each DNA construct using GeneJammer reagent (Agilent Technologies, Santa Clara, CA, USA), and were visualized by live-cell confocal imaging 36 h after transfection. All reagents for the cell cultures were obtained from Invitrogen (Waltham, MA, USA).

### Mn^2+^-quenching assay

Details of the principles and the procedure involved in the Mn^2+^-quenching assay are described elsewhere (Supplementary Fig. 22)^33^,^34^,^35^. Briefly, 0.5 mM Mn^2+^ was added to the extracellular medium of C2C12 skeletal myotubes that were loaded with fura-2-acetoxymethyl ester (AM) after TG-induced SR Ca^2+^ storage depletion without extracellular Ca^2+^ (by 50 μM BAPTA-AM), and the Mn^2+^-quenching of intracellular fura-2 fluorescence was measured at 360 nm (fura-2 excitation wavelength)^33^. The decay of the intracellular fura-2 fluorescence following the addition of Mn^2+^ was expressed as the percent decrease in the intracellular fura-2 fluorescence per unit time.

### Immunoblot assay, immunocytochemistry, or co-immunoprecipitation assay

Fully differentiated mouse primary skeletal myotubes on differentiation day 5 were solubilized, and the solubilized lysate (5 or 10 μg of total protein) was subjected to SDS-PAGE (8, 10, or 12% gel) and immunoblot assay, as previously described^12^,^32^,^39^,^46^,^47^. Anti-RyR1, anti-SERCA1a, anti-CSQ, anti-CaM1, anti-MG29, anti-MG53, anti-JP1, anti-JP2, anti-GFP, anti-myc, and anti-HA antibodies were obtained from Thermo Scientific Inc. (Rockford, IL, USA). Anti-TRPC1, anti-TRPC3, anti-TRPC4, and anti-TRPC6 antibodies were obtained from Alomone Laboratories (Jerusalem 9104201, Israel). Anti-DHPR, anti-Orai1, anti-STIM1, and anti-α-actin antibodies were obtained from Abcam (Cambridge, MA, USA). Immunocytochemistry using anti-GFP, anti-myc, Cy3-conjugated anti-mouse antibodies, and FITC-conjugated anti-rabbit secondary antibodies (Jackson ImmunoResearch, West Grove, PA, USA) was carried out, as described previously^19^,^39^,^46^,^49^. For co-immunoprecipitation assay, a triad vesicle sample (30 μg of total proteins) was subjected to immunoprecipitation using anti-Orai1 or anti-MG53 antibody, and then immunoblot assay with various antibodies, as previously described^30^,^32^,^46^. In the case of co-immunoprecipitation assay using HEK293 cells, HEK293 cells were lysed with a RIPA buffer (150 mM NaCl, 5 mM EDTA, 1% Nonidet P-40, 20 mM Tris-HCl, pH 7.5), and 20 μg of total protein was incubated with anti-HA antibody overnight, as previously described^19^.

### Single-myotube Ca^2+^ imaging experiment

Single-myotube Ca^2+^ imaging experiments were performed, as previously described^12^,^32^,^39^,^46^,^47^. Fully differentiated mouse primary skeletal myotubes on 96-well plates were loaded with 5 μM fura-2-AM (for the measurement of resting cytosolic Ca^2+^ levels) or with 5 μM fluo-4-AM (for other measurements) in an imaging buffer (25 mM Hepes, pH 7.4, 125 mM NaCl, 5 mM KCl, 2 mM KH_2_PO_4_, 2 mM CaCl_2_, 6 mM glucose, 1.2 mM MgSO_4_, and 0.05% BSA) at 37 °C for 45 min. The myotubes were transferred to an inverted stage microscope (Nikon Eclipse TS100, Melville, NY, USA) equipped with a 40X oil-immersion objective (NA 1.30). During single-myotube Ca^2+^ imaging, images of the myotubes were captured using a high-speed monochromator with a 75 W xenon lamp (FSM150Xe, Bentham Instruments, Verona, VA, USA) and a 12-bit charge-coupled device camera (DVC-340M-OO-CL, Digital Video Camera Company, Austin, TX 78744, USA). The data were displayed and analyzed using image acquisition and analysis software (High-Speed InCyt Im1 for fluo-4, and InCyt Im2 for fura-2, v5.29, Intracellular Imaging Inc, Cincinnati, OH, USA). Either caffeine or KCl was dissolved in the imaging buffer and applied via an auto-perfusion system (AutoMate Scientific, Berkeley, CA, USA). To measure the amount of releasable Ca^2+^ from the SR to cytosol, TG (2.5 μM, dissolved in DMSO, <0.05%) was manually applied to the myotubes in the absence of extracellular Ca^2+^ in order to avoid extracellular Ca^2+^ entry. DMSO (0.05%) alone had no effect on the release of Ca^2+^. For the measurement of SOCE, the SR Ca^2+^ storage was depleted with TG (2.5 μM) in the absence of extracellular Ca^2+^, and once the cytosolic Ca^2+^ level returned to the baseline, 2 mM Ca^2+^ was added to the myotubes to measure SOCE. To analyze the Ca^2+^ release obtained from the Ca^2+^ imaging experiments, the peak amplitude, which exhibited similar increases or decreases in peak areas, was considered. For long-term Ca^2+^ releases such as SOCE and TG responses, the areas under the curves were analyzed. All reagents for Ca^2+^ imaging experiments were obtained from Sigma-Aldrich (St. Louis, MO, USA).

### Statistical analysis

The results are presented as the mean ± S.E. for the number of myotubes shown in the parenthesis in Table 1 or the legends of Figures. An unpaired t-test (GraphPad InStat, v2.04, GraphPad Software, La Jolla, CA, USA) was used to compare the differences among groups. The differences were considered to be significant at *p* < 0.05.

## Additional Information

**How to cite this article**: Ahn, M. K. *et al*. Mitsugumin 53 regulates extracellular Ca^2+^ entry and intracellular Ca^2+^ release via Orai1 and RyR1 in skeletal muscle. *Sci. Rep.* **6**, 36909; doi: 10.1038/srep36909 (2016).

**Publisher’s note**: Springer Nature remains neutral with regard to jurisdictional claims in published maps and institutional affiliations.

## Supplementary Material

Supplementary Information

## Figures and Tables

**Table 1:** Properties of the mouse primary skeletal myotubes expressing full-length MG53 or PRY-SPRY.

		Vector	Full-length MG53	PRY-SPRY
KCl response		1.00 ± 0.06 (76)	0.75 ± 0.08 * (58)	0.81 ± 0.09 * (75)
Caffeine response		1.00 ± 0.08 (76)	0.72 ± 0.08 * (58)	0.83 ± 0.07 * (75)
Resting [Ca^2+^]_cytosol_		82.85 ± 7.30 (72)	110.75 ± 9.61 * (72)	106.08 ± 9.55 * (72)
Releasable Ca^2+^ from the SR		1.00 ± 0.07 (86)	1.02 ± 0.07 (76)	1.01 ± 0.08 (79)
SOCE	Ca^2+^-free for 6 min	1.00 ± 0.11 (87)	1.34 ± 0.09 * (60)	1.27 ± 0.09 * (86)
	Ca^2+^-free for 7 min	1.00 ± 0.09 (96)	1.32 ± 0.08 * (64)	1.28 ± 0.12 * (67)
	Ca^2+^-free for 8 min	0.78 ± 0.11 (162)	1.06 ± 0.09 * (179)	0.99 ± 0.10 * (166)

The values, except for those of resting Ca^2+^ levels and SOCE, were normalized to the mean value of those from vector controls. The values are presented as the mean ± S.E. for the number of myotubes shown in the parentheses. For SOCE measurement, the values were normalized to the mean value of those from vector controls at Ca^2+^-free for 6 min. *Significant difference compared with the Vector controls (*p* < 0.05).
