# Supplementary material for: Mitsugumin 53 regulates extracellular Ca2+ entry and intracellular Ca2+ release via Orai1 and RyR1 in skeletal muscle
Source: Sci Rep. 2016 Nov 14;6:36909. doi: 10.1038/srep36909 (PMC5107933; doi:10.1038/srep36909)
Supplement: Supplementary Information [file srep36909-s1.pdf]

# Supplementary Information

## Mitsugumin 53 regulates extracellular $Ca^{2+}$ entry and intracellular $Ca^{2+}$ release via Orai1 and RyR1 in skeletal muscle

**Mi Kyoung Ahn[1], Keon Jin Lee[1], Chuanxi Cai[2], Mei Huang[1], Chung-Hyun Cho[3], Jianjie Ma[4, *], and Eun Hui Lee[1, *]**

[1]Department of Physiology, College of Medicine, The Catholic University of Korea, 222 Banpo-daero, Seocho-gu, Seoul 06591, Republic of Korea
[2]Center for Cardiovascular Sciences, Department of Molecular and Cellular Physiology, Albany Medical College, 43 New Scotland Avenue, Albany, New York 12208, USA
[3]Department of Pharmacology, College of Medicine, Seoul National University, 103 Daehak-ro, Jongno-gu, Seoul 110-799, Republic of Korea
[4]Department of Surgery, Davis Heart and Lung Research Institute, The Ohio State University, 360 W. 12th Ave, Columbus, Ohio 43210, USA

[*]**Co-correspondence**: **Eun Hui Lee** (ehui@catholic.ac.kr); **Jianjie Ma** (jianjie.ma@osumc.edu)

**Supplemental Figure 1.** The full-length blots for Orai1 and MG53 in Figure 1b (upper panel).

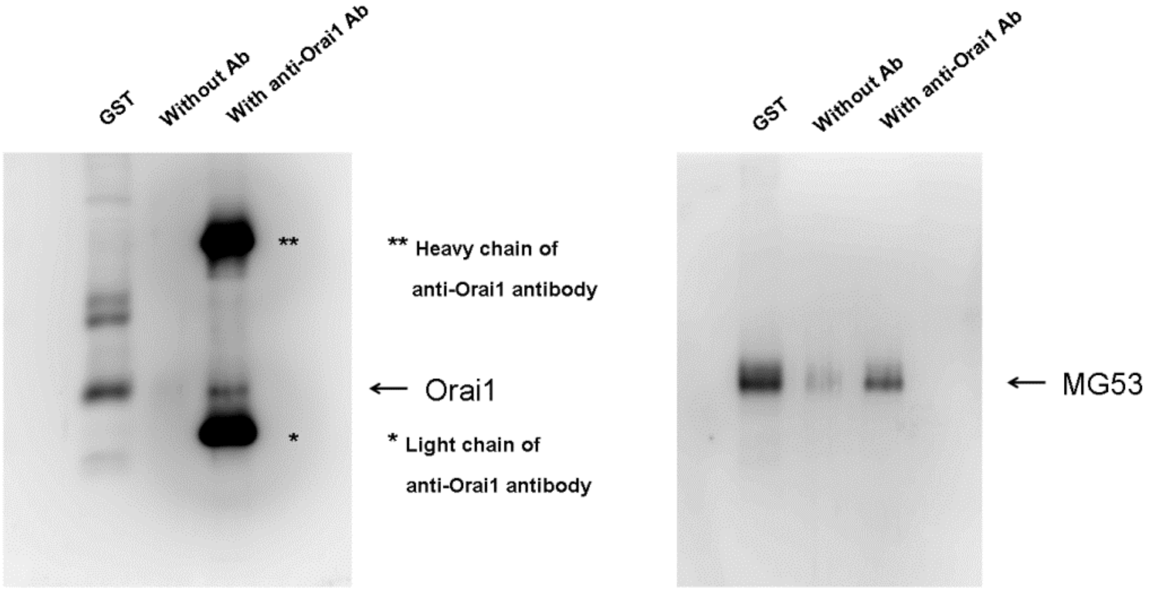


**Supplemental Figure 2.** The full-length blots for Orai1 and MG53 in Figure 1b (lower panel).

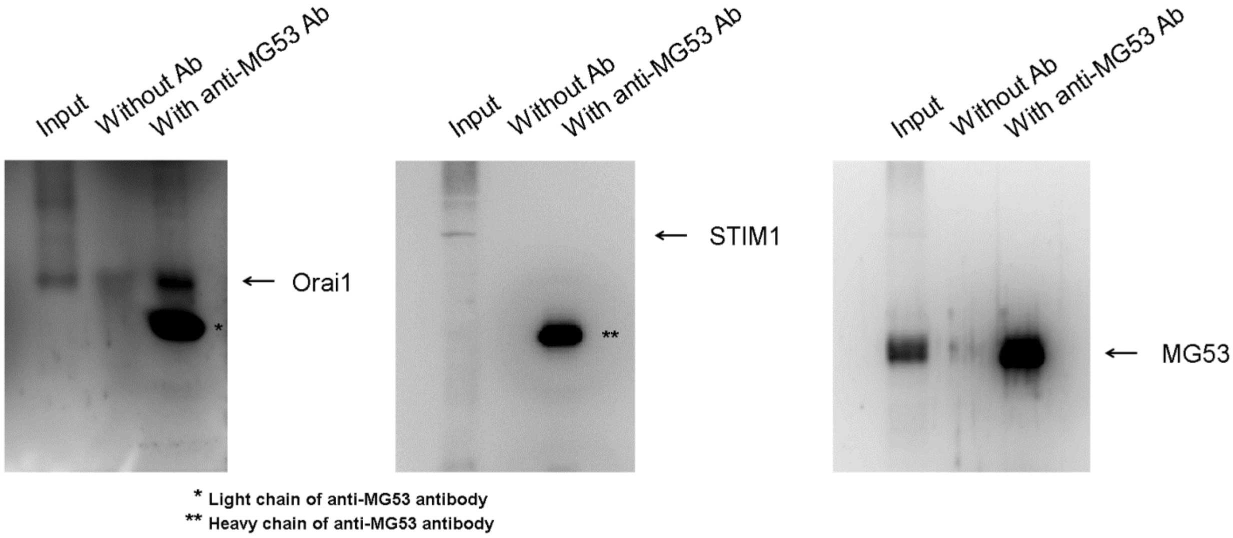

**Supplemental Figure 3.** The full-length blot for Orai1 in Figure 2c.

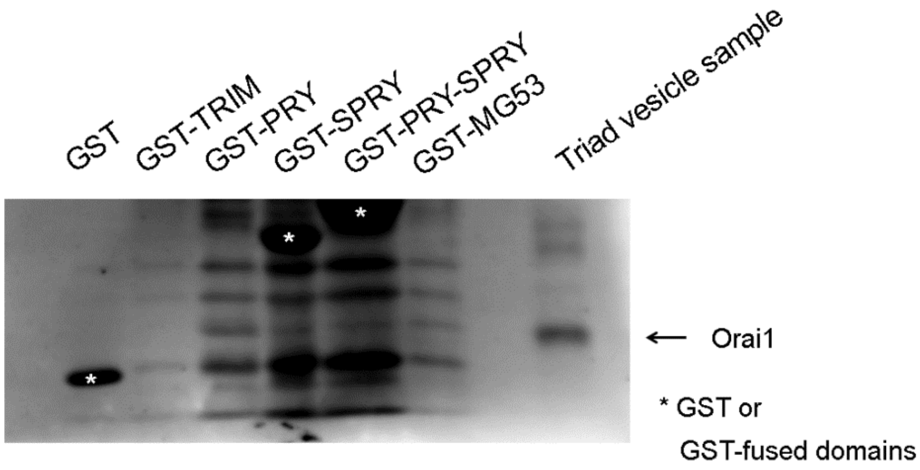


**Supplemental Figure 4.** The full-length blot for Orai1 in Figure 5a.

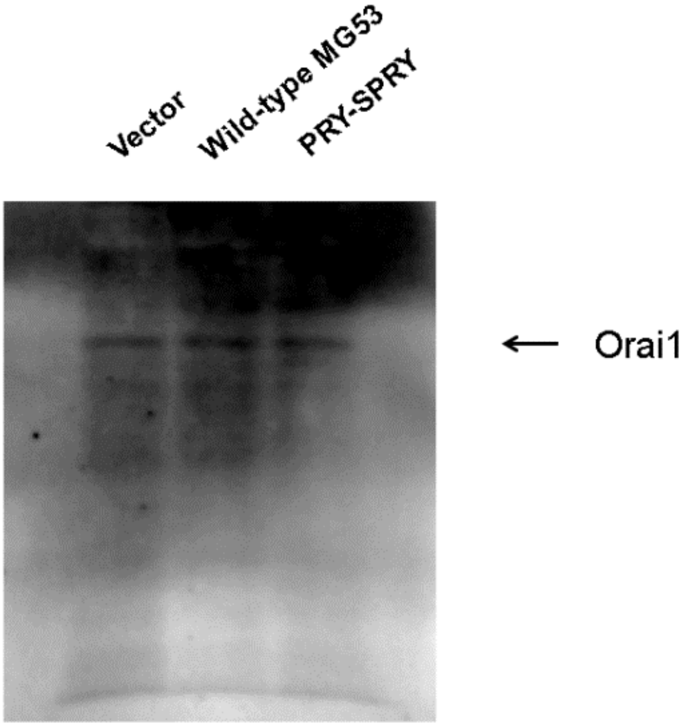

**Supplemental Figure 5.** The full-length blot for STIM1 in Figure 5a.

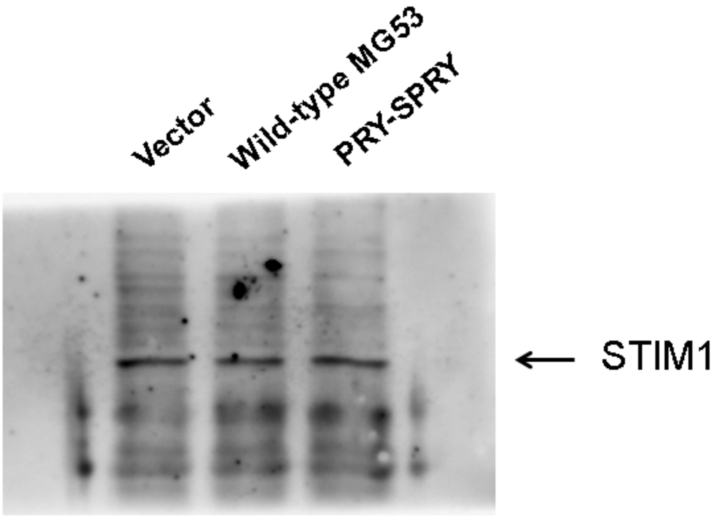


**Supplemental Figure 6.** The full-length blot for TRPC1 in Figure 5a.

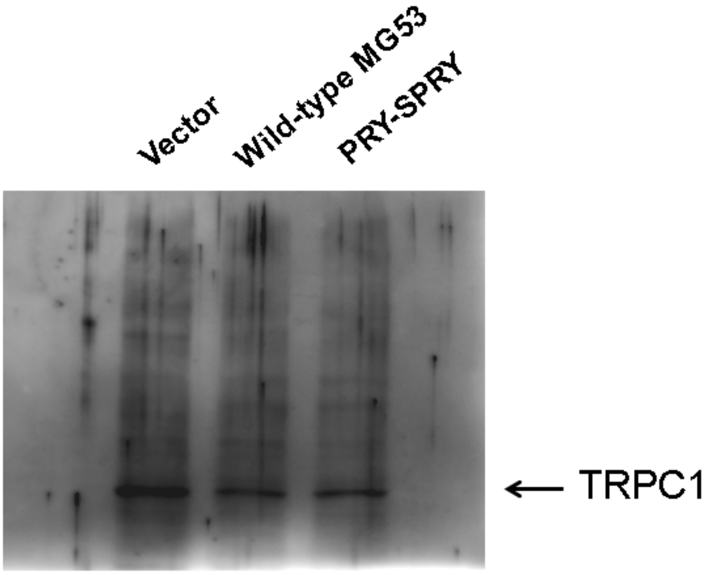

**Supplemental Figure 7.** The full-length blot for TRPC3 in Figure 5a.

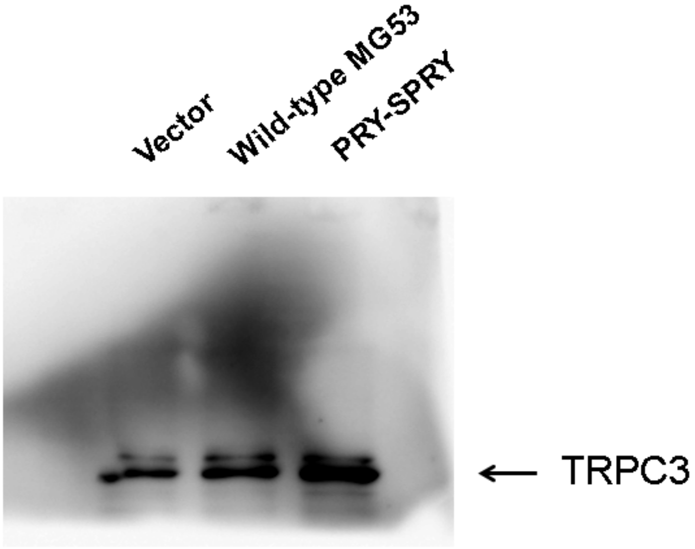


**Supplemental Figure 8.** The full-length blot for TRPC4 in Figure 5a.

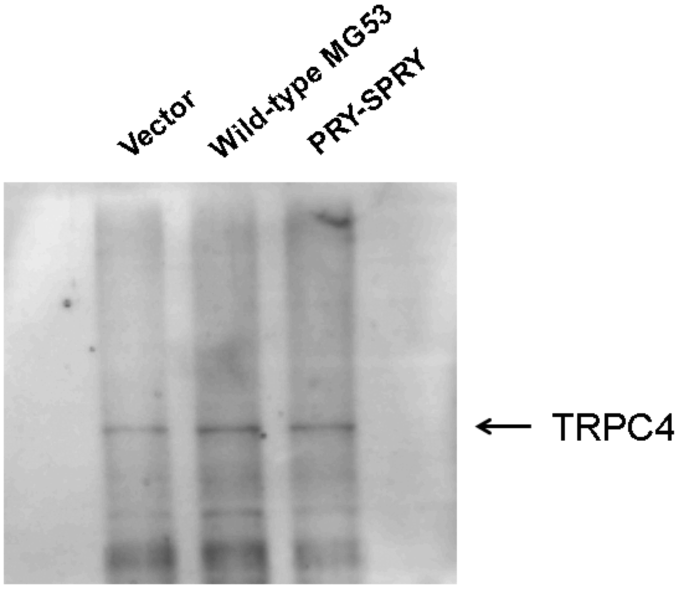

**Supplemental Figure 9.** The full-length blot for TRPC6 in Figure 5a.

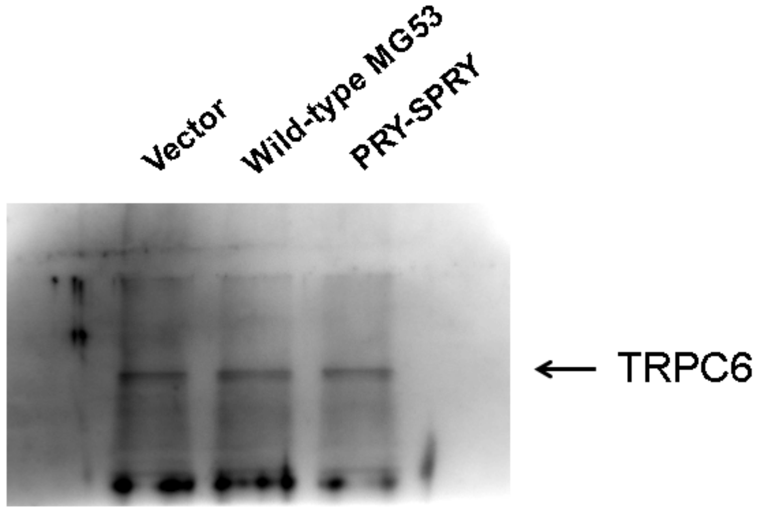


**Supplemental Figure 10.** The full-length blot for α-actin in Figure 5a.

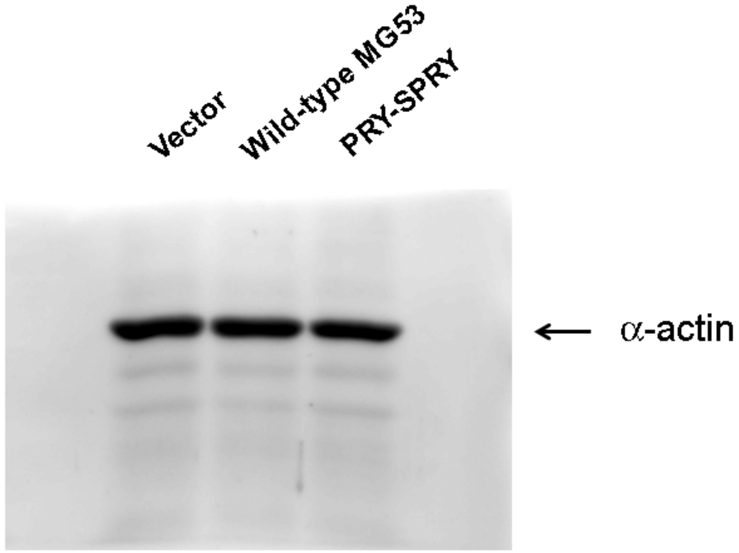

**Supplemental Figure 11.** The full-length blots in Figure 5c.

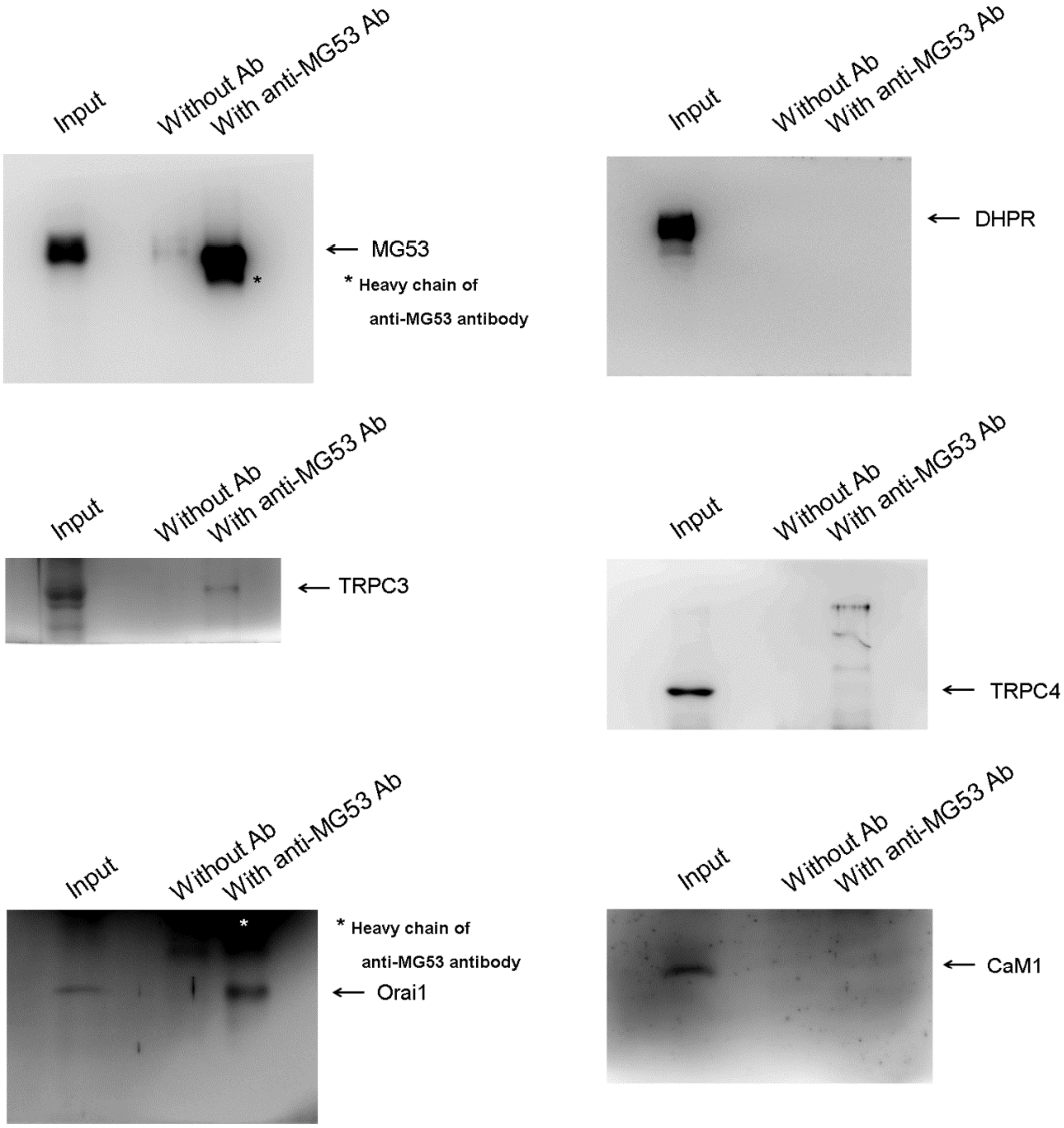

**Supplemental Figure 12.** The full-length blot for RyR1 in Figure 7a.

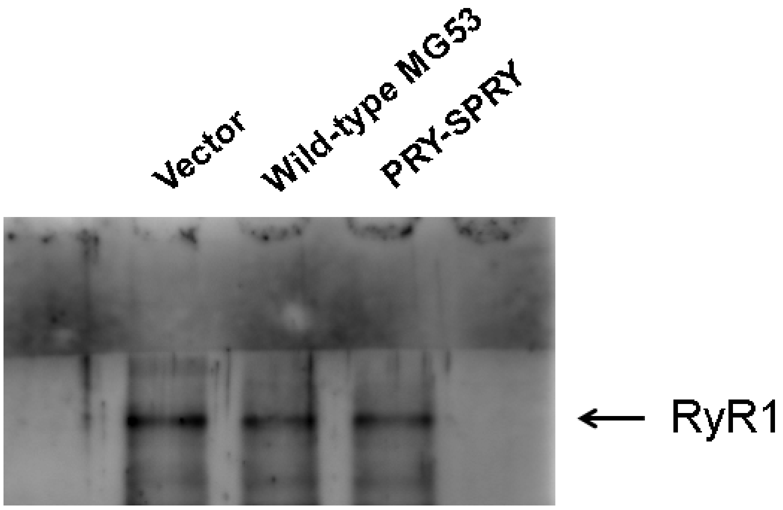


**Supplemental Figure 13.** The full-length blot for DHPR in Figure 7a.

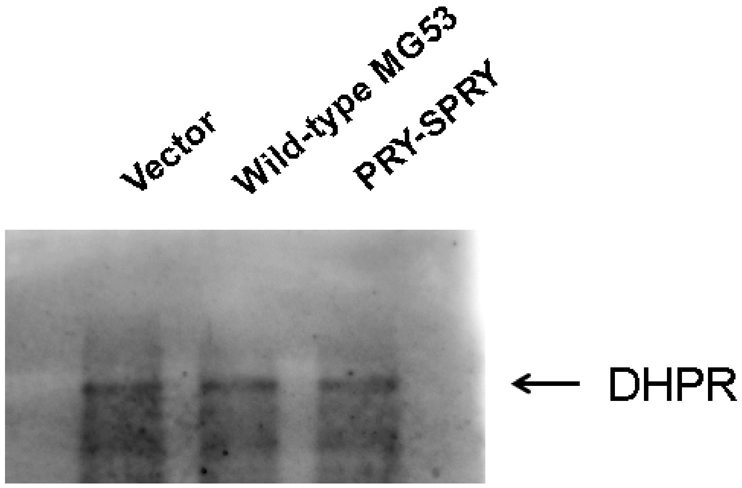

**Supplemental Figure 14.** The full-length blot for SERCA1a in Figure 7a.

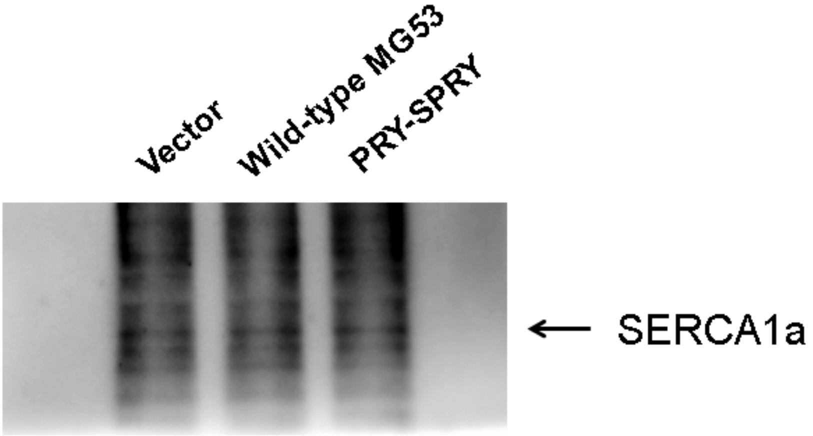


**Supplemental Figure 15.** The full-length blot for JP1 in Figure 7a.

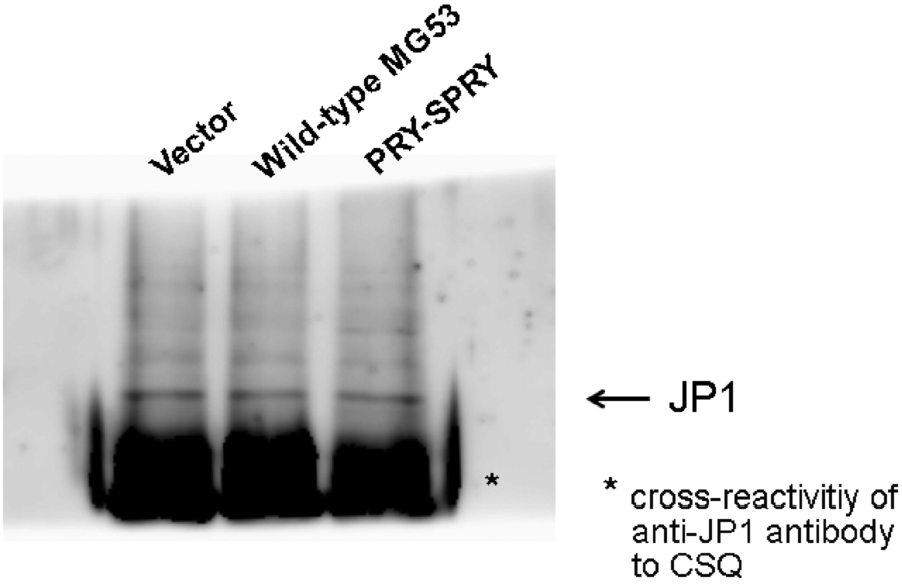

**Supplemental Figure 16.** The full-length blot for JP2 in Figure 7a.

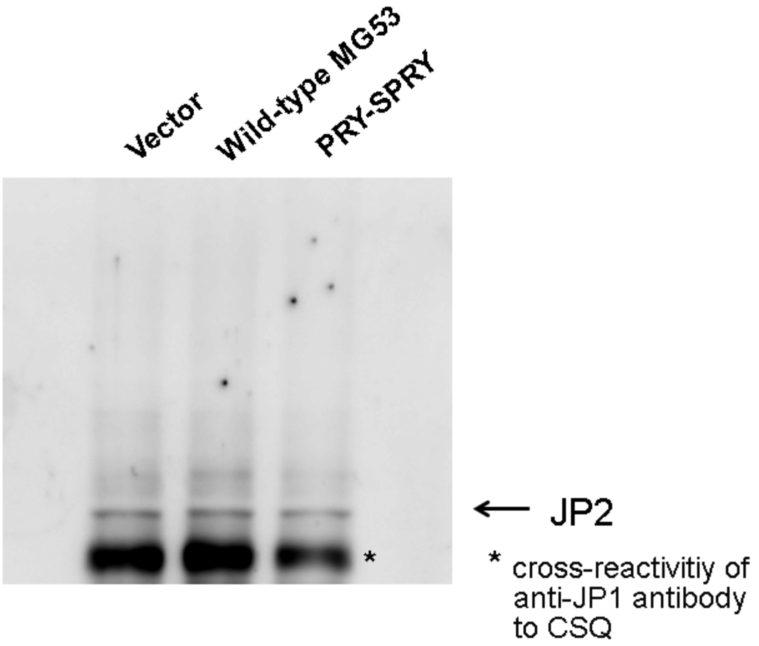


**Supplemental Figure 17.** The full-length blot for CSQ in Figure 7a.

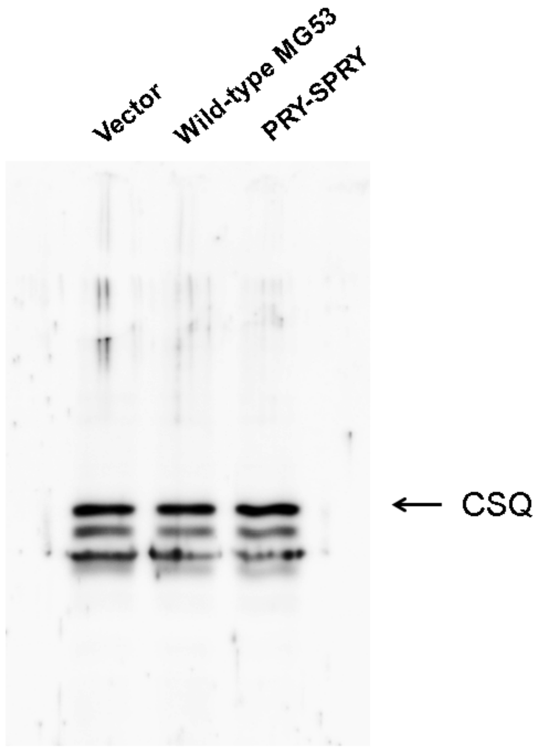

**Supplemental Figure 18.** The full-length blot for MG53 in Figure 7a.

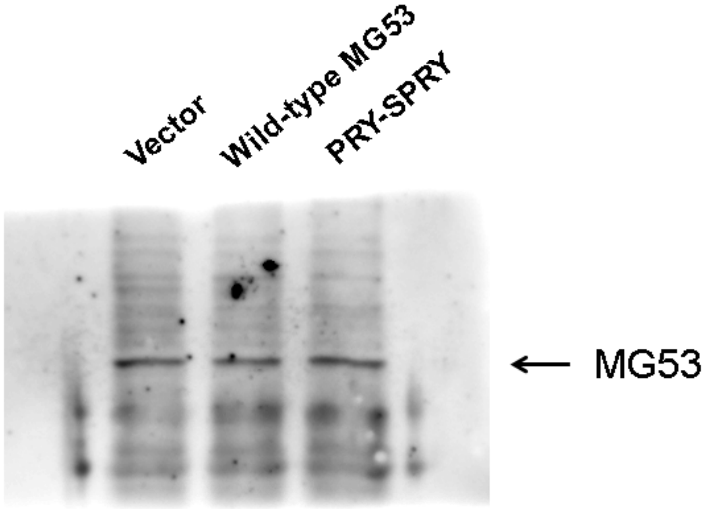


**Supplemental Figure 19.** The full-length blot for MG29 in Figure 7a.

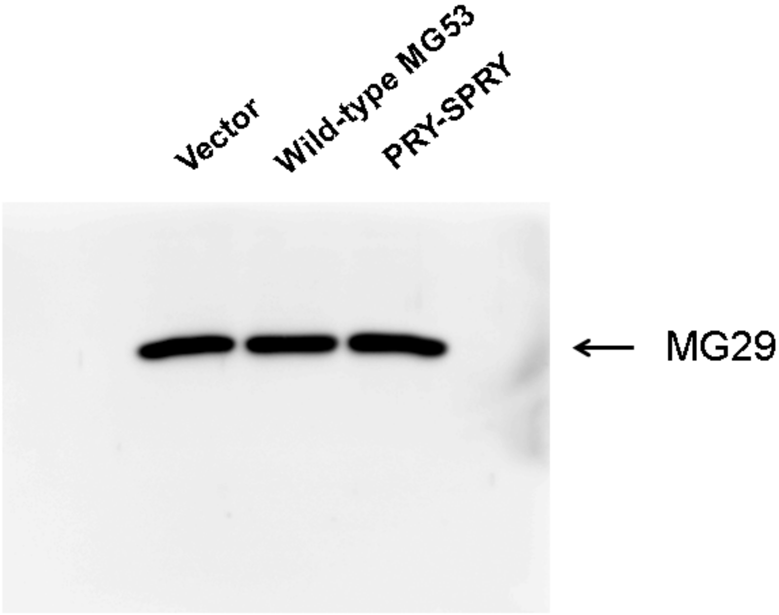

**Supplemental Figure 20.** The full-length blots for CaM1 in Figure 7a. Blots were obtained from three different periods of exposure time.

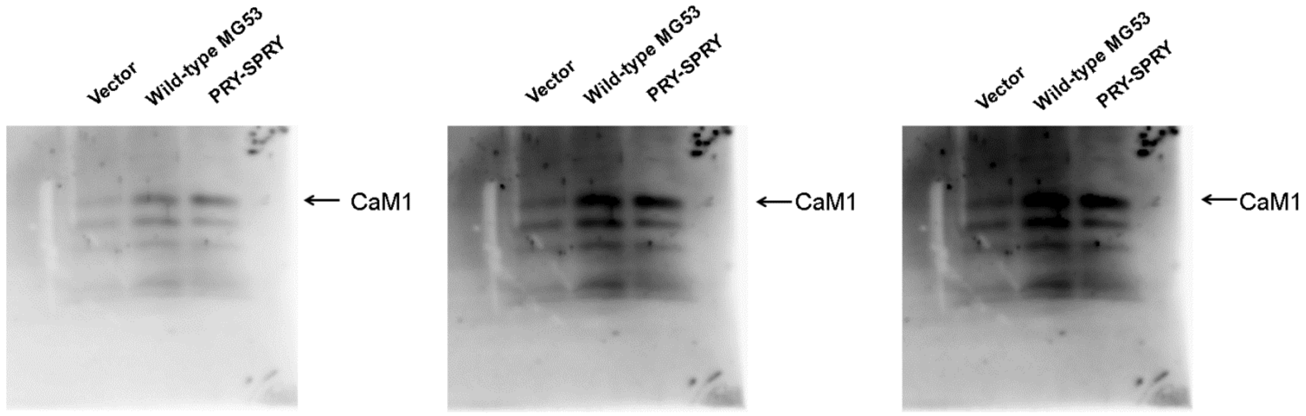


**Supplemental Figure 21.** The full-length blot for α-actin in Figure 7a.

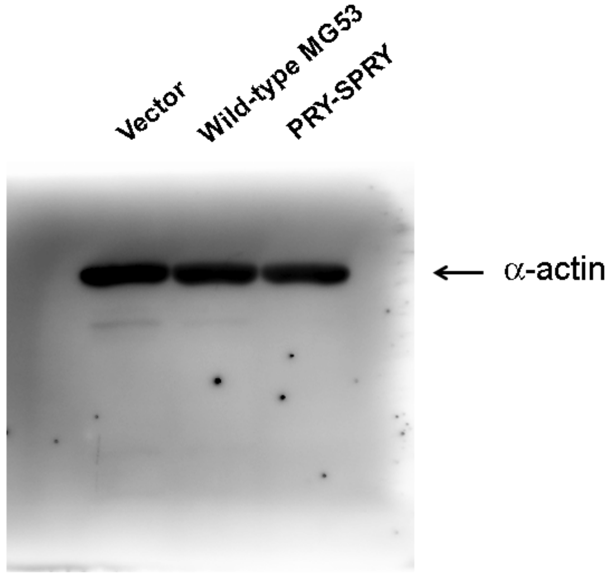

## **Supplemental Figure 22.** $Mn^{2+}$ quenching assay.

A $Mn^{2+}$ quenching assay measured the unidirectional SOCE from intracellular $Ca^{2+}$ release and $Ca^{2+}$ extrusion[1-3]. $Mn^{2+}$ permeates into cells via the SOCE mechanism while it is impervious to both the surface membrane extrusion processes and E/SR uptake by $Ca^{2+}$ pumps due to its very high affinity with intracellular fura-2. $Mn^{2+}$ with a $Ca^{2+}$-chelator such as BAPTA is added to the bath solutions (i.e., extracellular space) of cells loaded with fura-2. In this condition, extracellular $Ca^{2+}$ is chelated by BAPTA, and the depletion of E/SR $Ca^{2+}$ induces the entry of extracellular $Mn^{2+}$ into the cells instead of $Ca^{2+}$. As a result, the quenching of intracellular fura-2 fluorescence occurs with the entry of $Mn^{2+}$. The quenching of intracellular fura-2 fluorescence by $Mn^{2+}$ reflects the $Ca^{2+}$ entry via the SOCE mechanism. The decreased rate of intracellular fura-2 fluorescence intensity by $Mn^{2+}$-quenching is measured. A steeper slope indicates a more active SOCE, while a shallower slope means a less active SOCE. The decrease rate (i.e., the rate of $Mn^{2+}$ influx) is determined from the variable (i.e., slope) of a linear equation obtained from the linear fitting of the traces for the initial 10 seconds (i.e., the initial slope rather than the slower component from the late stages of the traces).

[1] Pan, Z., Zhao, X. & Brotto, M. Fluorescence-based measurement of store-operated calcium entry in live cells: from cultured cancer cell to skeletal muscle fiber. *Journal of visualized experiments : JoVE*, **60**, pii: 3415. doi:10.3791/3415 (2012).

[2] Shin, D. W. *et al.* A retrograde signal from calsequestrin for the regulation of store-operated $Ca^{2+}$ entry in skeletal muscle. *J Biol Chem* **278**, 3286-3292, doi:10.1074/jbc.M209045200 (2003).

[3] Pan, Z. *et al.* Dysfunction of store-operated calcium channel in muscle cells lacking mg29. *Nat Cell Biol* **4**, 379-383, doi:10.1038/ncb788 (2002).
